# Preliminary validation of a brief PROM assessing psychological distress in patients with malignant mesothelioma: The mesothelioma psychological distress tool—Patients

**DOI:** 10.3389/fpsyg.2022.974982

**Published:** 2022-11-25

**Authors:** Fanny Guglielmucci, Michela Bonafede, Danila Azzolina, Alessandro Marinaccio, Isabella Giulia Franzoi, Enrica Migliore, Carolina Mensi, Elisabetta Chellini, Elisa Romeo, Federica Grosso, Antonella Granieri

**Affiliations:** ^1^Department of Philosophy, Communication and Performing Arts, University of Roma Tre, Rome, Italy; ^2^Occupational and Environmental Medicine, Epidemiology and Hygiene Department, Italian Workers’ Compensation Authority (INAIL), Rome, Italy; ^3^Department of Environmental and Preventive Sciences, University of Ferrara, Ferrara, Italy; ^4^Department of Psychology, University of Turin, Turin, Italy; ^5^COR Piedmont, Unit of Cancer Epidemiology, University of Turin and CPO-Piedmont, Turin, Italy; ^6^COR Lombardy, Epidemiology Unit, Fondazione IRCCS Ca’ Granda, Ospedale Maggiore Policlinico and University of Milan, Milan, Italy; ^7^COR Tuscany, Cancer Prevention and Research Institute, Unit of Environmental and Occupational Epidemiology, Florence, Italy; ^8^COR Lazio, Department of Epidemiology, Lazio Regional Health Service, Rome, Italy; ^9^Mesothelioma Unit, Azienda Ospedaliera SS Antonio e Biagio e Cesare Arrigo, Alessandria, Italy

**Keywords:** asbestos, cancer, mental health, mesothelioma, patient-reported outcome measures, psychological distress, psycho-oncology, posttraumatic stress disorder

## Abstract

**Objective:**

Psychological suffering in malignant mesothelioma (MM) differs from that in other cancers because of its occupational etiology, and we aimed to develop specific patient-reported outcome measures to assess it.

**Methods:**

We used a multi-method prospective observational multicentric study (*N* = 149), and a preliminary questionnaire validation was performed through a Bayesian approach.

**Results:**

Item analysis showed a good internal consistency and reliability (Cronbach alpha = 0.79 [95% CI = 0.74–0.93]. Twenty of the 41 initial items were selected as posterior 95% highest density interval factor loading standardized effect size fell outside of the region of practical equivalence. Bayesian exploratory factor analysis showed a two-factor structure: (1) Trauma-related reactions (TR, 13 items) and (2) Claim for justice (CJ, 7 items), confirmed by the Bayesian confirmatory factor analysis. Latent factors were poorly correlated (Posterior median: 0.13; 95% CI = −0.079 to 0.323). The 90% root mean square error of approximation posterior median was 0.04 [90% CI = 0.03–0.58]; the 90% chi-square posterior median was 242 [90% CI = 209–287].

**Conclusion:**

Psychological suffering in MM patients implies negative cognitive, emotional, and somatic reactions related to the traumatic impact of the disease and the need to obtain justice through economic compensation. Our findings provide preliminary evidence that the Mesothelioma Psychological Distress Tool-Patients could be a promising and reliable instrument to assess MM patients’ psychological distress.

## Introduction

Malignant Mesothelioma (MM) is rare aggressive cancer related to asbestos exposure, which encompasses an interplay of medical, psychological, social and legal domains. The clinical facets of the disease (e.g., highly burden symptomatology, long-lasting period before symptoms development, lack of effective treatments, short life expectancy, work-related etiology) (Moore et al., 2009) play an important role in understating its mental representation and psychosocial dimensions. Recently, increasing attention has been focused on a patient-centered approach which emphasizes the need for a comprehensive assessment of MM’s impact and MM care (Granieri, 2015), and the urge for developing patient-reported outcome measures (PROMs) has been largely described by researchers and clinicians (Novello et al., 2016; Bonafede et al., 2018).

PROMs are self-reported questionnaires assessing symptom burden, personal experience of care, and patients’ health-related quality of life. They are used in clinical research and practice with relevant implications for the healthcare and reimbursement systems (Black, 2013). In principle, PROMs could be general or disease-specific. Nonetheless, the latter allows the detection of specific conditions and their impact in a population of interest (Weldring and Smith, 2013).

Literature review have suggested a specific characterization of psychological suffering in MM patients (Ball et al., 2016; Noonan, 2017; Bonafede et al., 2018) due to several aspects: limited prognosis and a lack of effective treatment options; distress and fatigue linked with the compensation process; the guilt and shame that often arises after being exposed to asbestos in the workplace; an inability to understand or recognize the different health risks.

In particular, the psychological distress in MM patients is mostly related to the occupational etiology of the disease (Noonan, 2017). Indeed, different from other cancers, a certain responsibility in the onset of MM could be traced to corporations and industrial activities (Agudo et al., 2000; Bonafede et al., 2020). The occupational etiology of MM influences attributional processes, as an external culprit can be identified, shaping the way patients and family members emotionally and behaviorally react to MM (Granieri et al., 2020).

MM patients often feel betrayed for having been exposed to asbestos: they blame governments for having allowed the use of such a harmful material and for not having adequately protected their health and that of family members (Obata, 2011) and they experience anger toward those who put their lives at risk (Guglielmucci et al., 2014; Granieri, 2018). To restore their perceived control over a powerless condition and minimize their responsibility for becoming ill and having exposed their beloved ones to invisible environmental health threats, MM patients’ aggressive stance may take the form of claims and class action lawsuits to receive economic compensation (Guglielmucci, 2016; Sherborne et al., 2020). Nonetheless, the legal journey to obtain justice and compensation may become a source of stress for these patients (Riblier-Dehen et al., 2019), who is still emotionally involved with and grateful to their past employers and corporations which provided them a job and livelihood for many years (Clayson et al., 2005; Arber and Spencer, 2013).

Despite existing measures adapted for MM patients (Hollen et al., 2004), available tools are focused especially on lung cancer (Koller et al., 2020; Quaife et al., 2021) and do not seem to detect the complex medical, psychological, social, and legal dynamics involved in MM. For these reasons, we aimed to develop a brief PROM (the Mesothelioma Psychological Distress Tool—Patients, MPDT-P) to evaluate the specific profile of psychological suffering in this population.

As MM is rare cancer, whose prevalence is less than 1% of that of all cancers (The Portal for Rare Diseases and Orphan Drugs, 2022), clinical research often involves small sample sizes, as in our case. Statistical analyses in small sample sizes are a challenge for researchers (Smid et al., 2020). In some cases, frequentist approaches, consider asymptotic statistics which may not be valid for small sample sizes (Rupp et al., 2004). Within this general framework, a Bayesian approach for assessing items’ quality in detecting the psychological construct and exploring the factor structure of the questionnaire, could be a suitable approach, especially for a limited sample size. (Spiegelhalter et al., 1994). As no prior information was available, we used uninformative priors, which are suggested especially for inferences conducted on small sample sizes (Spiegelhalter et al., 1994; Bernardo and Ramón, 1998; Azzolina et al., 2021).

## Materials and methods

Recommendations for developing valid and responsive evidence-based tools suggest a careful literature review process (Kline, 1986) and a multi-method approach considering patient perspectives and the experience of clinicians (Revicki et al., 2008). According to these recommendations, we developed a multi-method prospective observational multicentric study with the following steps: (i) systematic literature review to identify the main constructs which characterize the psychological suffering of MM patients (Bonafede et al., 2018); (ii) operationalization of the identified constructs in facets and subfacets; (iii) test construction and item development; (iv) qualitative evaluation of an item pool through focus groups with experts and the target population; (v) quantitative evaluation of the item pool through a Subject Matter Expert (SME) methodology. Participants were recruited through the Italian National Mesothelioma Registry (Registro Nazionale dei Mesoteliomi – ReNaM), a national epidemiological surveillance system based on regional centers (Centri Operativi Regionali – COR) that searches for MM cases actively and investigates the modalities of asbestos exposure (Marinaccio et al., 2018). Seventeen interviewers of the involved COR (Tuscany, Piedmont, Lombardy, and Lazio) were trained by psychological staff (March–June 2018). Participants were recruited from the ReNaM register (INAIL, 2018; Marinaccio et al., 2018) of the regions involved in the project, they were contacted by telephone to request adhering to the project and interviewed in person. Patients who gave their written consent were enrolled in the study and consecutively administered by MPDT-P. A separate form for collection of sociodemographic data and medical data was developed. The data were collected confidentially and treated in aggregate form. A total of 707 individuals were recruited and 149 of them decided to participate in our study.

### Statistical analysis

#### Data description

Continuous data were described in terms of the first, median, and second quartile. Categorical variables were reported in terms of absolute and relative frequencies.

#### Missing data imputation

**Figure 1:**
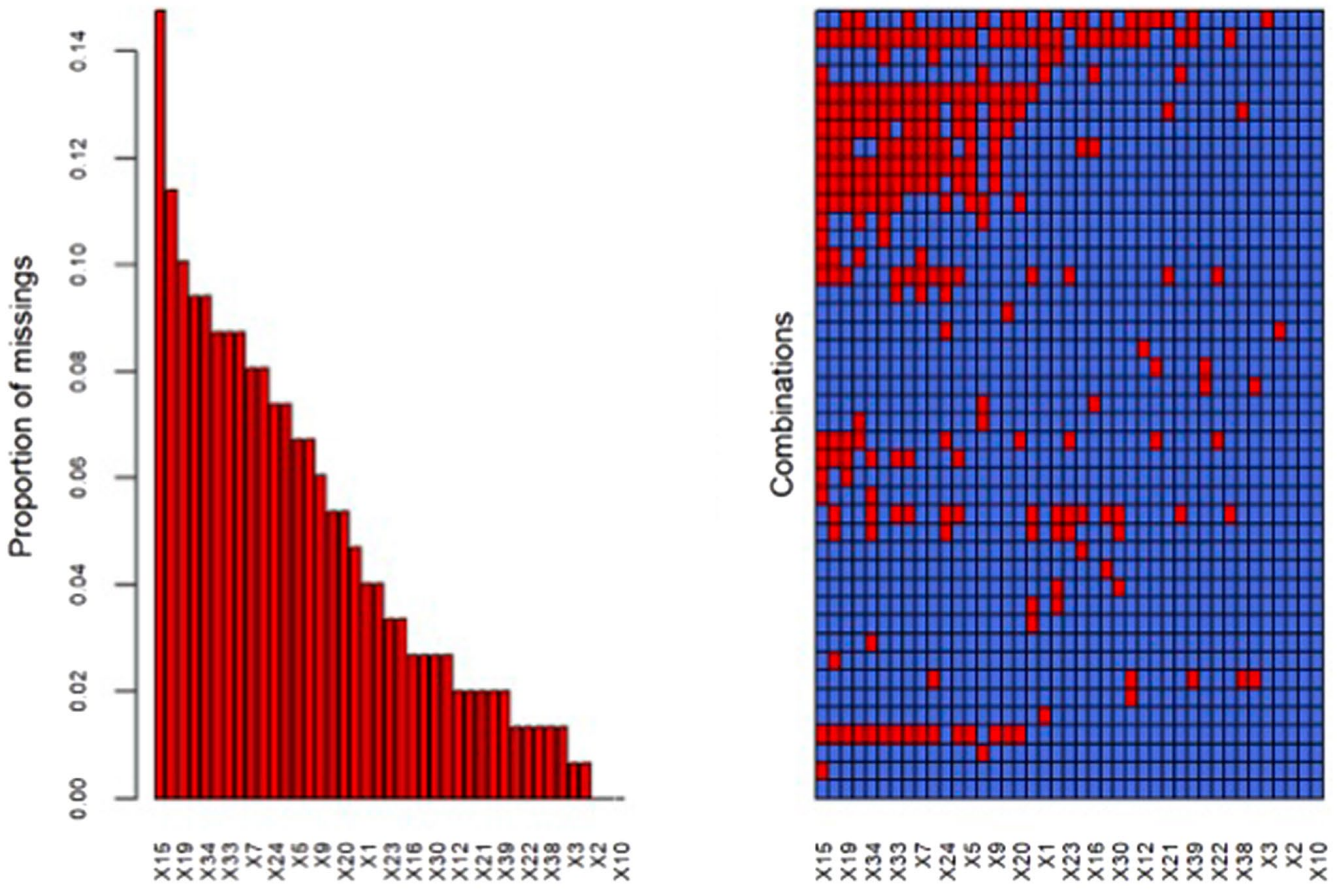
Proportion of missing data acoording to questionaire item (panel A) and missing pattern plot according to responders and items (panel B).

A common issue faced with multivariate data with missing values is whether the missing data are missing completely at random (MCAR); that is, whether missingness depends on the variables in the data set. The proposed method of assessing this is to compare the means of each variable between groups defined by whether other variables in the data set are missing or not. The method involves many correlated statistics for testing MCAR, resulting in multiple-comparison problems. In this research, we consider a single global test statistic for MCAR that uses all of the available data as reported in the literature (Little, 1988). The percentage of missing data is reported in Figure 1, together with the missingness pattern across observation and variables. Multiple chained equation imputation (MICE) was implemented for MCAR data (van Buuren and Groothuis-Oudshoorn, 2011). Alternatively, the missing not-at-random imputation models for a multiple imputation solution would have been considered (Galimard et al., 2018).

#### An early validation effort *via* a Bayesian approach

The preliminary questionnaire validation process, fully conducted in a Bayesian framework, consists of a three-step procedure:

- An item analysis was carried out to identify the overall and item-specific questionnaire reliability.
- The Bayesian Exploratory Factor Analysis (BEFA) was conducted to identify a subset of latent factors explaining the questionnaire structure together with the items having considerable importance (loadings) on the identified latent dimension.
- The Bayesian Confirmatory Factor Analysis (BCFA) was also conducted as verification of the BEFA-identified latent item-dimension construct.

The Bayesian reliability analysis has been also performed on the subsections and the overall final questionnaire Statistical details on the questionnaire validation procedure have been included in the Supplementary Material.

Analyses were performed in R 3.4.2 (R Development Core Team R, 2015) by using RBtest (Rouzinov and Berchtold, 2022), mice (van Buuren and Groothuis-Oudshoorn, 2011), BayesRel (Pfadt et al., 2021), BayesFM (Piatek, 2020), and Blavaan (Merkle and Rosseel, 2018) packages.

## Results

### Data description and missing data imputation

The study sample (Table 1) is composed of 149 subjects 54% males, having a median age of 71 years, with medium educational attainment (i.e., a high school diploma, 33%). The leading enrolling center (Table 1) was A.O. SS Antonio e Biagio e Cesare Arrigo (AL) (49%), followed by the Santo Spirito Hospital, Casale Monferrato (16%).

**Table 1:** Patient details. Continuous variables are synthesized in terms of first, median, and second quartile; categorical variables are reported in terms of absolute and relative frequencies.

Variable	*n*	I, median, and II quartile
Age	136	65.75/71.00/77.00
		
Variable	n	%
**Gender**		
Female	62	46
Male	73	54
Total	135	100
**Center**		
Santo Spirito Hospital, Casale Monferrato (AL)	24	16
A.O. SS Antonio e Biagio e Cesare Arrigo (AL)	73	49
COR Piedmont	9	6
IRCCS Ca′ Granda Ospedale Maggiore Policlinico (MI)/ COR Lombardy	10	7
COR Novara	3	2
COR Lazio	8	5
COR Tuscany	21	14
Total	148	100
**Education attainment**		
3 years	4	3
5 years	28	21
6 years	1	1
8 years	45	33
8 years + professional qualification	1	1
13 years	38	28
16 years	1	1
18 years	17	13
Total	135	100

Concerning the missing data distribution (Figure 1) a greater percentage (15%) of missing questionnaire responses was observed on item X15, followed by X19 and X34. Variables other than the first two do not present more than 10% of missing data.

The Little’s global test evidenced that the missing data among all variables in the database may be defined as MCAR. Therefore, a MICE model imputation was considered, because it is suitable to impute MCAR and MAR data (van Buuren and Groothuis-Oudshoorn, 2011).

### Questionnaire validation

#### Item analysis

The posterior median estimate (Supplementary Figure S1) for the Cronbach alpha was 0.79 [95% CI = 0.74–0.93]. The posterior probability that alpha should be higher than 0.70 and smaller than 0.90 was 0.99, indicating an appropriate questionnaire reliability measure (Galimard et al., 2018). The dropped-item reliability analysis revealed that even if every single item was dropped from the questionnaire, the median alpha level would remain lower than or equal to the global 0.79 alpha (Table 2). For this reason, no items were dropped in this phase.

**Table 2:** Bayesian individual item reliability statistics posterior mean and 95% credible intervals (CIs) Cronbach’s *α* (if item dropped).

Item	Posterior Median	Lower 95% CI	Upper 95% CI
X1	0.780	0.730	0.829
X2	0.782	0.735	0.833
X3	0.783	0.734	0.832
X4	0.775	0.724	0.826
X5	0.779	0.729	0.828
X6	0.783	0.731	0.830
X7	0.795	0.745	0.838
X8	0.787	0.737	0.833
X9	0.782	0.732	0.830
X10	0.782	0.733	0.830
X11	0.787	0.736	0.831
X12	0.778	0.723	0.824
X13	0.786	0.737	0.833
X14	0.783	0.732	0.830
X15	0.780	0.727	0.826
X16	0.784	0.731	0.829
X17	0.781	0.732	0.832
X18	0.788	0.739	0.834
X19	0.775	0.722	0.824
X20	0.782	0.730	0.829
X21	0.791	0.742	0.835
X22	0.779	0.729	0.829
X23	0.777	0.724	0.825
X24	0.774	0.723	0.824
X25	0.783	0.728	0.827
X26	0.783	0.734	0.832
X27	0.778	0.725	0.826
X28	0.773	0.723	0.826
X29	0.781	0.731	0.830
X30	0.780	0.728	0.828
X31	0.777	0.722	0.824
X32	0.775	0.721	0.823
X33	0.768	0.713	0.818
X34	0.780	0.727	0.827
X35	0.792	0.743	0.836
X36	0.784	0.735	0.832
X37	0.780	0.735	0.833
X38	0.781	0.727	0.826
X39	0.788	0.738	0.833
X40	0.780	0.731	0.830
X41	0.781	0.728	0.828

#### BEFA analysis

The posterior probability on the optimal number of factors of identification identifies two latent dimensions as the most suitable solution to represent the questionnaire subspace (Supplementary Figure S4).

**Figure 2:**
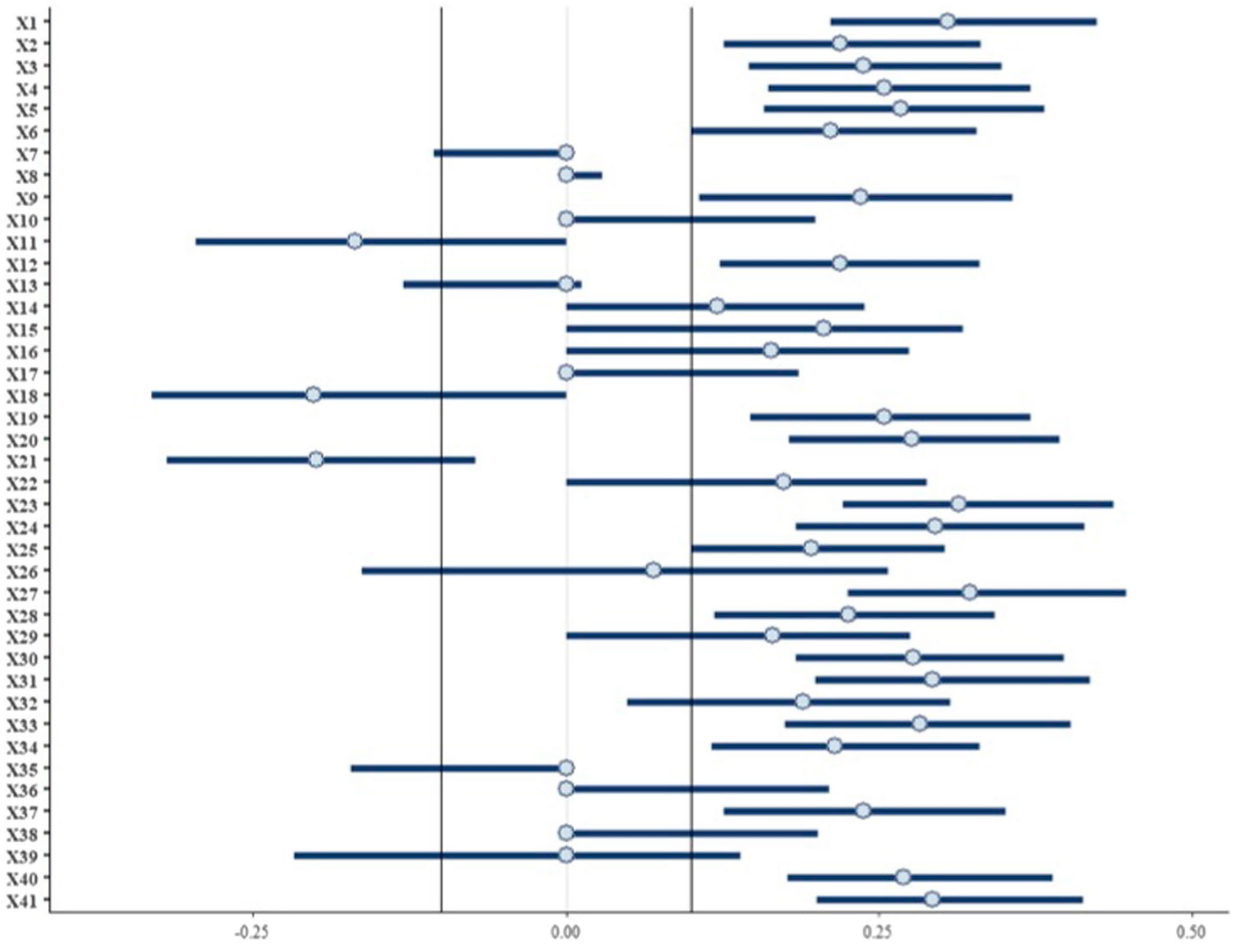
Bayesian Exploratory Factor Analysis 95% highest density interval (HDI) posterior estimate on the standardized factor loading. The region of practical equivalence area is identified through black lines.

The standardized BEFA posterior 95% highest density interval (HDI) factor loading standardized effect size is reported in Figure 2 together with the HDI-region of practical equivalence (ROPE) limits. Items lie outside the ROPE area.

Among the 20 ROPE survived items, 13 of them are saturated on the first dimension (Table 3) (X1, X2, X3, X4, X12, X20, X23, X27, X30, X31, X34, X40, and X41) and 7, on the second latent dimension (X5, X9, X19, X24, X28, X33, and X37) (item specification in the appendix).

**Table 3:** BEFA 95% HDI cross-loading on identified factors.

Item	Factor	Loadings 95% CI
X1	1	0.3 [0.21–0.42]
X2	1	0.22 [0.13–0.33]
X3	1	0.24 [0.15–0.35]
X4	1	0.25 [0.16–0.37]
X12	1	0.22 [0.12–0.33]
X20	1	0.28 [0.18–0.39]
X23	1	0.31 [0.22–0.44]
X27	1	0.32 [0.23–0.45]
X30	1	0.28 [0.18–0.4]
X31	1	0.29 [0.2–0.42]
X34	1	0.21 [0.12–0.33]
X40	1	0.27 [0.18–0.39]
X41	1	0.29 [0.2–0.41]
X5	2	0.27 [0.16–0.38]
X9	2	0.24 [0.11–0.36]
X19	2	0.25 [0.15–0.37]
X24	2	0.3 [0.18–0.41]
X28	2	0.23 [0.12–0.34]
X33	2	0.28 [0.17–0.4]
X37	2	0.24 [0.13–0.35]

#### BCFA analysis

**Figure 3:**
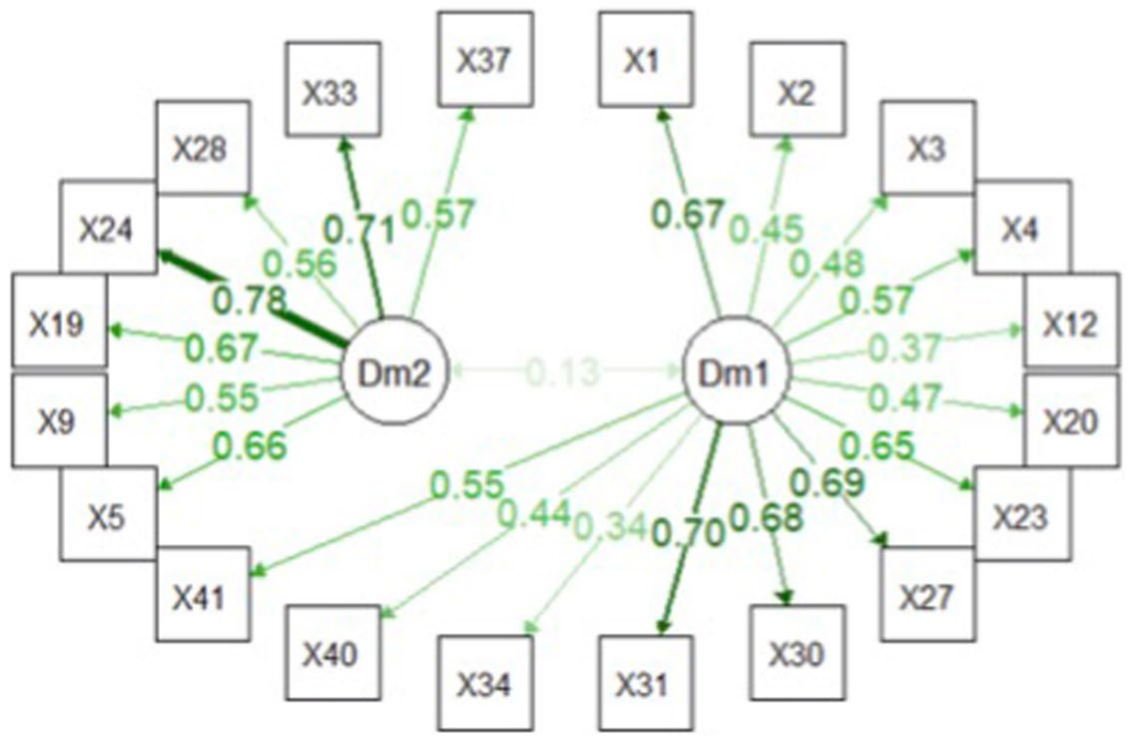
Bayesian Confirmatory Factor Analysis (BCFA) factor path. The standardized factor loading on the first (Dml) and second (Dm2) dimensions have been reported, together with the correction among latent factor 0.13 [95% CI = −0.079 to 0.323].

The BCFA was performed on the 20 ROPE BEFA survival items. The structural model path (Figure 3) identifies two separate dimensions addressing separate constructs among them, poorly correlated with the CI bound crossing the zero value. The posterior median is 0.13 [95% CI = −0.079 to 0.323].

**Figure 4:**
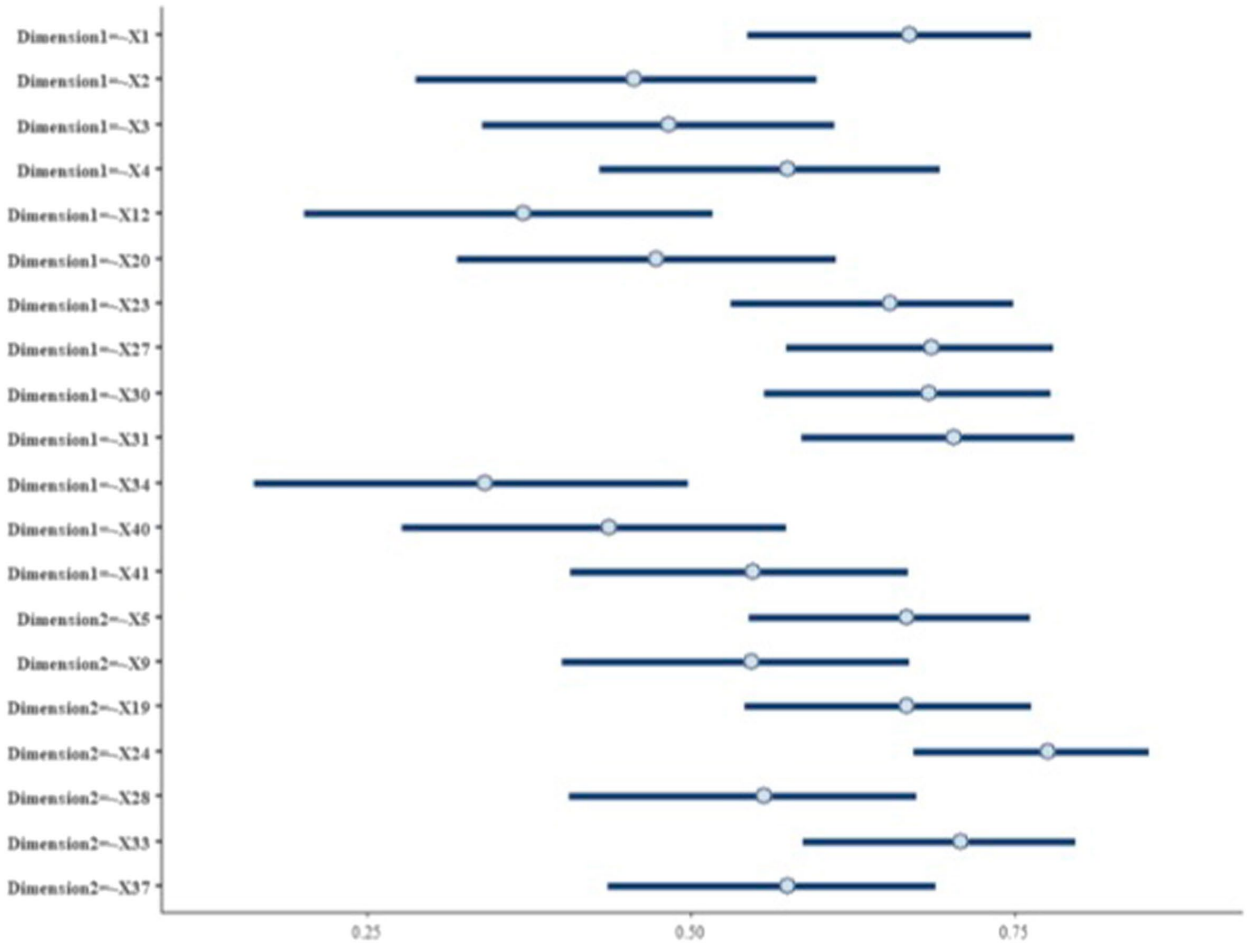
BCFA 95% HDI posterior estimate on the standardized factor loading. The latent dimensions identified on each item have been also reported on the Y-axis.

Concerning factor loadings (Figure 4), higher loadings on the first dimension are reported for items X27 and X31; items X33 and X24 present higher loadings on the second dimension (Figure 3).

All the HDI intervals for the BCFA-standardized loadings on the 20 BEFA survival items lie outside the ROPE region, indicating that the construct domain structure previously identified by the BEFA analysis is confirmed in the BCFA approach (Figure 4).

#### Model fitting and diagnostics

The root mean square error of approximation (RMSEA) posterior probability has been reported in Supplementary Figure S5. The probability that RMSEA lies below the optimal fit threshold of 0.06 was 95%. The 90% RMSEA posterior median was 0.04 [90% CI = 0.03–0.58]. For chi-squared, the posterior median was 242 [90% CI = 209–287].

The performance metric indicate a suitable fit. The trace plots for parameters across MCMC computation have been reported in Supplementary Figures S6–S8. The diagrams showed the absence of pattern across iterations indicating an appropriate model convergence for Item Analysis, BEFA analysis, and BCFA analysis.

#### Reliability analysis of the final questionnaire

The posterior median estimate for the overall Cronbach alpha concerning the overall questionnaire composed of the selected items is 0.83 [95% CI = 0.77–0.86]. The result indicates an improved internal consistency in comparison with the original instrument. The reliability performance is moreover satisfactory also for the items loading on the first factor with a median alpha of 0.84 [95% CI = 0.80–0.87] and the second factor with a median alpha of 0.82 [95% CI = 0.78–0.86].

The split-half reliability analysis identifies a median Cronbach alpha across computations of 0.81 with a median difference in alpha between the split half of the sample of 0.03 [95% CI = 0.004–0.06], indicating a suitable internal consistency result.

## Discussion

We present the development of the first specific PROM aimed at detecting the psychological suffering of MM patients, providing preliminary evidence of its psychometric proprieties.

Bayesian analyses showed the good internal consistency and reliability of the questionnaire (Cronbach alpha = 0.79 [95% CI = 0.74–0.93), and a 20-item two-factor structure. Based on the content of the items (Appendix A), we labeled the factors as (1) Trauma-Related Reactions (TR) and (2) Claims for Justice (CJ).

The first factor (13 items) covers a plethora of negative cognitive (e.g., intrusive thoughts and nightmares), emotional (e.g., depressive conditions of hopelessness and loss of interest, death anxieties, and shame) and bodily reactions (e.g., sweating, tachycardia, nausea, and diarrhea). These negative reactions may occur not just at diagnosis (Arber and Spencer, 2013) but also during treatment, and when adjusting to life afterward (Bonafede et al., 2018). Individual reactions to MM may vary, from more adaptive and resilient ones (Hughes and Arber, 2008; Guglielmucci, 2016) to the development of severe psychopathological conditions (Dooley et al., 2010; Arber and Spencer, 2013; Granieri et al., 2017). Clinically, they ought to be considered as self-conscious and unconscious reactions to the traumatic onset of MM in the individual’s life, related to underlying dysfunctional mental processes.

In-depth qualitative studies of the subjective experience of patients living with MM found that they felt ashamed, blamed, and stigmatized for their illness (Hughes and Arber, 2008; Borgogno et al., 2015; Franzoi et al., 2015; Granieri et al., 2017). Even decades after asbestos exposure, self-blaming and “*an unpleasant feeling with accompanying beliefs that one should have thought, felt, or acted differently*” (i.e., guilt) may persist (Borgogno et al., 2015; Guglielmucci et al., 2018a; Bonafede et al., 2022).

The most severe responses to MM include the activation of dysfunctional defense mechanisms (Lebovits et al., 1983; Borgogno et al., 2015; Granieri et al., 2017) and trauma-related dissociation (Granieri, 2015; Guglielmucci et al., 2018b), which seem to be related to a pre-existing maladaptive personality functioning (Granieri, 2018; Granieri et al., 2018a). Therefore, trauma-related beliefs and emotions moved away from consciousness may intrude the mind of MM patients as thoughts or nightmares related to the trajectory of the disease or may be expressed *via* somatic symptoms (Granieri, 2013, 2015, 2017), increasing the risk of developing post-traumatic symptoms (Lee et al., 2009).

The second factor (7 items) reflects a reactive/reparative position characterized by feelings of anger and betrayal for having been exposed to a harmful pollutant, along with the desire to obtain economic compensation for it.

The literature has extensively shown that when individuals become aware of having developed MM because of the negligence of governments or private companies’ profit, they often feel angry and betrayed (Clayson et al., 2005; Granieri, 2013; Guglielmucci et al., 2014; Testoni et al., 2019). They perceive their disease as “unjust” (Hughes and Arber, 2008), caused by people who have intentionally ignored asbestos warnings, and adopted inadequate safety procedures because they did not even care for their health and safety (Granieri, 2015). It is not unusual that the perception of having been irreparably damaged leads to claims for compensation (Di Basilio et al., 2021; Guglielmucci, 2016).

Seeking compensation for asbestos exposure has complex moral and normative meanings (Guglielmucci et al., 2015) and allows the obtention of public recognition for what happened (Granieri, 2015; Granieri et al., 2018b). In this process, moral emotions (e.g., guilt and shame) play an important role, and guilt seems to be a motivational process to enact reparative behaviors (Pillayre, 2021). From this perspective, the desire to be compensated underpins the need to restore a violated trust and sense of justice (Bernardo and Ramón, 1998; Tangney et al., 2007), and to overcome one’s guilt and responsibility for having developed MM and having exposed one’s family to such a health threat (Guglielmucci et al., 2014; Thébaud-Mony, 2003).

### Conclusion

We provide significant research progressing our clinical understanding and assessment of psychological suffering in MM patients.

Our results (i.e., the posterior of the present analysis) could become the prior distribution for future research developments, by gradually reducing the uncertainty on the final inference (Rupp et al., 2004). Moreover, they also emphasize the utility of adopting a trauma-focused framework for conceptualizing MM-related psychological pain, as already proposed in the literature (Hawley and Monk, 2004; Guglielmucci et al., 2014; Granieri, 2016; Di Basilio et al., 2021; Bonafede et al., 2022).

Future research should be aimed to cross-validate our results to test the predictive ability of MPDT-P to detect subjects at risk of developing severe psychopathologies.

Identifying underlying mental dynamics related to the traumatic impact of the disease is a crucial but hard process, worsened by the reluctance of MM patients to openly share their thoughts and express how they feel (Chapple et al., 2004; Granieri et al., 2017; Guglielmucci et al., 2018a; Girgis et al., 2019; Warby et al., 2019; Walker et al., 2021; Bonafede et al., 2022). Integrating the use of MPDT-P in clinical practice should help practitioners overcome this issue and obtain a quick and reliable picture of MM-related specific suffering. Such results should be used to collect important information, detect risky conditions, and promptly activate public health-integrated programs (Granieri, 2015). Indeed, compared with other psychological assessment tools, this questionnaire helps to investigate the differences related to the occupational and environmental contexts that caused the disease. Different work histories of different contaminated communities may, in fact, lead to different psychological patterns. The relevance of the present study consists in the possibility to assess the specific patient’s suffering and provide tailored psychological care.

### Study limitations

Our sample could be considered small concerning common statistical guidelines, but it included a substantial proportion of the Italian population (20% of total MM cases in the regions of Italy involved in the project according to INAIL). It also has to be noted that clinical research on traumatized populations is quite difficult to address and often implies working with small samples. For these reasons, we have applied a Bayesian approach which provides a set of methodological tools and a broader philosophical framework particularly useful for studying and understanding psychological trauma. Someone could argue that the main limitation of this study is the lack of external-criterion validity assessment. Thus, before applying inadequate representation of psychometric modeling, it was crucial to develop a theoretical representation of the construct following a mixed clinical-conceptual and data-driven approach.” Other research is needed to assess the generalizability of the proposed instrument by extending the reliability analysis, not only by repeating the surveys over time (test–retest reliability) but also by varying the sample of respondents for the confirmatory analysis.

However, the sample considered in this research is representative of a wide set of patients referring to several Italian centers covering the Centre-Northern Italy zone. In this research framework, the EFA and CFA performed on the same data allow a preliminary assessment of the instrument properties. Moreover, we may also consider that different data set may yield different test outcomes. Conducting both EFA and CFA on the same data reduces such a possibility, especially in a preliminary validation phase. The model derivation in one-half of the data and validation in another set could be a solution that has been not considered given the sample size which is not large as compared to the number of parameters to be estimated.

A content comparison with other general measuring distress tools could be also performed but it should be considered that, that the available questionnaires do not seem to detect the complex medical, psychological, social, and legal dynamics involved in MM.

Another limitation is represented by the low response rate; the reported responses are the 16% of the overall sample size as provided by the study protocol. Such limitation is related to the critical health status of the patients involved in the study; this aspect constitutes a limitation to the full study compliance.

## Data availability statement

The raw data supporting the conclusions of this article will be made available by the authors, without undue reservation.

## Ethics Statement

The study was conducted according to the guidelines of the Declaration of Helsinki and approved by the Ethical Committee of the Azienda Ospedaliera SS Antonio e Biagio e Cesare Arrigo (AVPM-14/11/2014).

## Author contributions

FGu, MB, and AG: conceptualization. FGu and DA: data curation, formal analysis, and methodology. FGu, MB, AM, IF, EM, CM, EC, ER, and FGr: investigation. AG: project administration and supervision. FGu, MB, AM, IF, EM, CM, EC, ER, FGr, and AG: resources. FGu and MB: writing—original draft. FGu, MB, IF, and AG: writing—review and editing. All authors contributed to the article and approved the submitted version.

## Funding

This work was supported and partially funded by INAIL Research Plans 2019–2021, project BRIC n. 55.

## Conflict of interest

The authors declare that the research was conducted in the absence of any commercial or financial relationships that could be construed as a potential conflict of interest.

## Publisher’s note

All claims expressed in this article are solely those of the authors and do not necessarily represent those of their affiliated organizations, or those of the publisher, the editors and the reviewers. Any product that may be evaluated in this article, or claim that may be made by its manufacturer, is not guaranteed or endorsed by the publisher.
