# Supplementary material for: Preliminary validation of a brief PROM assessing psychological distress in patients with malignant mesothelioma: The mesothelioma psychological distress tool—Patients
Source: Front Psychol. 2022 Nov 25;13:974982. doi: 10.3389/fpsyg.2022.974982 (PMC9732528; doi:10.3389/fpsyg.2022.974982)
Supplement: Supplementary file 1 [file Data_Sheet_1.pdf]

Data di compilazione____________ Intervistatore ______________________________

Gentile paziente, Le chiediamo di indicare il suo grado di accordo/disaccordo con le affermazioni sotto riportate

1 Completamente in disaccordo

2 In disaccordo

3 D’accordo

4 Completamente d’accordo

Dopo la diagnosi di malattia

1	Mi sento privo di speranza	1	2	3	4
2	Ho sentito spesso una forte agitazione interna	1	2	3	4
3	Ho spesso incubi o brutti sogni	1	2	3	4
4	Da quando sono malato ho spesso attacchi di rabbia, anche per cose non importanti	1	2	3	4
5	Penso che l’azienda/le istituzioni avrebbero dovuto tutelare maggiormente i lavoratori/cittadini	1	2	3	4
6	Mi vergogno per aver esposto i miei cari a un rischio di salute	1	2	3	4
7	Nonostante abbia lavorato/vissuto in un luogo contaminato che mi ha esposto a un rischio di salute sento ancora un forte legame con l’azienda/con il territorio	1	2	3	4

8	Se mi sono ammalato è anche una mia responsabilità	1	2	3	4
9	È preciso dovere dell’azienda/delle istituzioni risarcirmi per il danno subito	1	2	3	4
10	Spesso evito di pensare di essere malato	1	2	3	4
11	Cerco di trovare sempre qualcosa di positivo nonostante la mia malattia	1	2	3	4
12	Mi sento/mi sono sentito una cavia	1	2	3	4
13	Mi capita spesso di pensare alle cose belle della vita	1	2	3	4
14	A volte gli effetti collaterali delle terapie sono peggiori della malattia stessa	1	2	3	4
15	È preciso dovere dell’azienda/delle istituzioni provvedere al sostentamento della mia famiglia ora che non posso più lavorare	1	2	3	4
16	Non mi fido di come mi stanno curando	1	2	3	4
17	Mi sento in colpa perché ho esposto i miei cari al rischio di ammalarsi	1	2	3	4
18	Nonostante sia ammalato mi concedo momenti di piacere	1	2	3	4
19	Se la mia azienda/le istituzioni mi avessero informato sui rischi forse non mi sarei ammalato	1	2	3	4
20	Mi vergogno per essermi ammalato di mesotelioma	1	2	3	4
21	Ho trovato nuovi modi per continuare a fare le cose che mi piacciono	1	2	3	4
22	Vorrei avere maggiori informazioni sulle cure disponibili incluse quelle sperimentali	1	2	3	4

23	Il solo ricordo della comunicazione della diagnosi mi causa disturbi fisici importanti (sudorazione, tachicardia, nausea, diarrea, etc...)	1	2	3	4
24	Sento un gran bisogno di giustizia per quello che mi è successo	1	2	3	4
25	Evito di parlare della mia malattia e delle emozioni che mi suscita	1	2	3	4
26	Parlo con gli operatori sanitari per comprendere meglio la mia situazione medica	1	2	3	4
27	La vita quotidiana mi sembra troppo difficile per poterla affrontare	1	2	3	4
28	Sono arrabbiato perché mi sono ammalato a causa del mio lavoro/a causa del luogo in cui vivevo	1	2	3	4
29	Cerco sostegno dalle persone che mi stanno vicino	1	2	3	4
30	Ho paura del futuro	1	2	3	4
31	Mi sento angosciato rispetto all’idea della morte	1	2	3	4
32	Mi torna in mente il momento in cui mi hanno detto che sono malato	1	2	3	4
33	Mi fa arrabbiare che nessuno mi abbia informato dei rischi di salute che correvo	1	2	3	4
34	Essermi ammalato nel luogo in cui lavoravo/vivevo mi crea imbarazzo	1	2	3	4
35	Nel complesso penso che la mia azienda/le istituzioni non mi abbiano poi trattato così male	1	2	3	4
36	Mi rimprovero di non essere andato via dal luogo di lavoro/dove vivo	1	2	3	4

37	Chi ha causato questa situazione deve essere punito	1	2	3	4
38	Cerco di fare più cose possibili durante la giornata per non pensare alla malattia	1	2	3	4
39	Affronto quello che viene, passo dopo passo	1	2	3	4
40	Davanti ai diversi trattamenti di cura provo un senso di confusione e non so cosa fare	1	2	3	4
41	Le cose che prima mi davano piacere adesso sono senza senso per me	1	2	3	4

Tempo di somministrazione: ____
